# Effects of Different Carbohydrate Level Diets on Growth Performance and Glycometabolism in Mandarin Fish (*Siniperca chuatsi*) Under Saline‐Alkali Stresses

**DOI:** 10.1155/anu/3315751

**Published:** 2026-05-21

**Authors:** Yufu Kong, Jinquan Fan, Yan Li, Erchao Li, Liqiao Chen, Xiaodan Wang

**Affiliations:** ^1^ School of Life Sciences, East China Normal University, Shanghai, China, ecnu.edu.cn; ^2^ East China Sea Fisheries Research Institute, Chinese Academy of Fishery Sciences, Shanghai, China, cafs.ac.cn

**Keywords:** carbohydrate utilization, glycometabolism, saline-alkali stress, *Siniperca chuatsi*

## Abstract

Saline‐alkali cultivation is a crucial approach to alleviate the fresh water crisis. However, prolonged saline‐alkali stresses might bring adverse effects on fish growth, health, and even death. This study evaluated the effects of different carbohydrate supplementation on growth performance, carbohydrate metabolism, antioxidant capacity, and tissue morphology of mandarin fish (*Siniperca chuatsi*) under saline‐alkali stress. Fish with an initial weight of 31.03 ± 0.12 g, were fed with three carbohydrate levels (8%, 13%, and 18%) in either a freshwater or a saline‐alkali water environment (salinity = 6, alkalinity = 0.7 g/L). Results indicated that fish cultured in saline‐alkali water performed a higher specific growth rate (SGR) and weight gain (WG). In addition, the activities of the glycolysis pathway, pentose phosphate pathway, and gluconeogenesis pathway were enhanced prominently under this stress. The growth performances were also improved significantly by the increased carbohydrate diets. However, the activities of amylase were inhibited with the increased carbohydrate intake, which impeded the process of glycometabolism. In contrast, the pentose phosphate pathway and gluconeogenesis pathway responded positively to dietary carbohydrate levels, especially under saline‐alkali stresses. Moreover, improving the carbohydrate level appropriately could significantly reduce the accumulation of malondialdehyde, promote the activities of superoxide dismutase, and total antioxidant capacity under saline‐alkali stress. Furthermore, the expression of insulin receptors showed an ascending tendency in freshwater, while opposite in saline‐alkali water with increased carbohydrate intake. In summary, an appropriate saline‐alkali environment was more conducive to the growth of mandarin fish. Moreover, 13% carbohydrate diets could enhance its growth performance in saline‐alkali environments. It was noteworthy that the effects of insulin on the regulation of carbohydrate metabolism might not be the same as normal conditions during the adaptation process in mandarin fish. The pentose phosphate pathway played a critical part in osmoregulation under saline‐alkali stress.

## 1. Introduction

With the rapid development of aquaculture and the shortage of freshwater resources, the trend of saline‐alkali water aquaculture is increasingly growing; [1–6]. However, prolonged saline‐alkali stress brought adverse effects on fish growth and healthy such as osmotic balance, ion toxicity, and oxidative stress, thereby impairing immune and death [7–12]. Consequently, enhancing the survival, adaptability, and growth performance of fish had emerged as a pivotal challenge in aquaculture development.

Under saline‐alkali stress, osmotic regulation was essential for fish to maintain physiological homeostasis [13–17]. It had been shown that carbohydrate metabolism was enhanced under saline‐alkali stress in various species to ensure energy availability and osmotic stability [18–20]. For instance, the gluconeogenesis pathway was activated under saline‐alkali stress in large yellow croaker (*Larimichthys crocea*), and similar results were observed in Mozambique tilapia (*Oreochromis mossambicus*) [20, 21]. In other research, fish supplemented with carbohydrates in diets demonstrated more active glycometabolism and better growth performance [22, 23]. These findings suggested that carbohydrate metabolism was critical for the growth and adversity adaptation of fish. Moreover, formulating a reasonable carbohydrate regulation strategy might be of great significance for promoting the adaptation capacity of fish under saline‐alkali stress.

In contrast to herbivorous and omnivorous fishes (e.g., grass carp, *Ctenopharyngodon idellus*, and common carp, *Cyprinus carpio*), whose tolerance to dietary carbohydrates reaches ~40%, carnivorous fishes generally exhibited lower carbohydrate tolerance, typically 20% below [24–26]. Limited tolerance of carbohydrate was ascribed to reduced α‐amylase activity, impaired glucose transport capacity, insufficient insulin secretion, and decreased insulin receptor numbers [24, 27–30], which seemingly cast a shadow over promoting salt‐alkali adaptability by increasing the carbohydrate level in feed. However, studies also demonstrated that carbohydrate metabolism in carnivorous fishes, like spotted seabass (*Lateolabrax maculatu*s), yellowfin seabream (*Acanthopagrus latus*) and large yellow croaker (*Larimichthys crocea*) were significantly activated under saline‐alkali water [21, 31, 32]. This means that the carbohydrate utilization in carnivorous fish might be improved by environmental stresses, providing us a direction for further exploring the changes in glycometabolism under saline‐alkali or saline stresses, as well as the adaptation strategies centered around carbohydrates.

Mandarin fish (*Siniperca chuatsi*), as an economic freshwater carnivorous species, have been proved to possess high economic value, lower carbohydrate tolerance (≤8%) and cultivated in saline‐alkali water with great potential [33–35]. However, limited research had been conducted on its carbohydrate metabolism and adaptive responses under saline‐alkali conditions [35, 36]. It is essential to investigate the regulatory pathways of carbohydrate metabolism in *S. chuatsi* under saline‐alkali conditions, as well as to assess the effects of different dietary carbohydrate levels on its metabolic response, stress resistance, and growth performance. Therefore, this study was aimed to provide a theoretical basis and technical support for enhancing the saline‐alkali adaptability of *S. chuatsi*, improving feed formulation, and increasing aquaculture productivity.

## 2. Materials and Methods

### 2.1. Experimental Diets

Diets were designed for three categories with different pregelatinized starch: 8% (NC), 13% (HC1), and 18% (HC2). The formulations and approximate composition of the three experimental diets are given in Table 1. All powder components were well mixed after being lifted twice through a 40‐mesh strainer. Then, the diet was extruded into a 2.5 mm pellet, air‐dried and stored at −20°C until use.

**Table 1:** Formulation and chemical composition of the experimental diets (%, dry matter).

Ingredients	Groups				
	NC	HC1		HC2	
Fish meal	45.00	45.00		45.00	
Casein	16.00	16.00		16.00	
Soybean protein concentrate	10.00	10.00		10.00	
Pregelatinized starch	8.00	13.00		18.00	
Fish oil	2.00	2.00		2.00	
Soybean oil	2.00	2.00		2.00	
Lecithin	2.00	2.00		2.00	
Vitamin premix^a^	0.50	0.50		0.50	
Mineral premix^b^	0.50	0.50		0.50	
Ca(H_2_PO_4_)_2_	0.50	0.50		0.50	
Choline chloride	0.50	0.50		0.50	
Dimethyl‐beta‐propiothetin	0.10	0.10		0.10	
Butylated Hydroxytoluene	1.00	1.00		1.00	
Sodium alginate	1.00	1.00		1.00	
Microcrystalline cellulose	10.90	5.90		0.90	
Chemical composition (%)					
Moisture	11.40	11.96		11.88	
Crude protein	44.57	44.10		44.26	
Crude lipid	11.56	11.01		10.79	
Crude ash	9.77	9.69		9.69	

^a^Vitamin premix (mg/kg of diet): vitamin A, 8000 IU; vitamin D3, 2000 IU; vitamin E, 200; vitamin K3, 20; vitamin B1, 30; vitamin B2, 50; vitamin B6, 20; vitamin B12, 0.2; pantothenic acid, 60; niacin acid, 200; vitamin C, 500; inositol, 600; biotin, 3.2; folic acid, 10. All of them were filled into rice bran.

^b^Mineral premix (mg/kg of diet): FeSO_4_•H_2_O, 500; ZnSO_4_•H_2_O, 500; CuSO_4_•5H_2_O, 40; MnSO_4_•H_2_O, 200; MgSO_4_•H_2_O, 2500; potassium iodide, 100; CoCl_2_•·6H_2_O, 100； potassium chloride, 250; Sodium Selenite, 70. All of them were filled into rice bran.

### 2.2. Animal and Experimental Design

The fish used in this study were obtained from the base of Guledeng Aquatic Products Breeding Center, Huzhou City, Zhejiang Province. Mandarin fish were acclimated for 2 weeks before experiments. 450 juvenile mandarin fish (31.03 ± 0.12 g) with similar physical fitness were screened and randomly assigned to eighteen 500‐L round barrels with 25 fish in each barrel. Fish were divided into six groups, three replicates each group, cultivated between saline‐alkali water (salinity = 6, was achieved by adding sea crystal (main component is sodium chloride); alkalinity = 0.7 g/L, was achieved by adding sodium bicarbonate) and fresh water, fed for three different level of carbohydrate (8%, 13%, and 18%), respectively. And we nominated them as FW + NC, FW + HC1, FW + HC2, SAW + NC, SAW + HC1, and SAW + HC2. During the 42 days culture experiment, fish were fed at 4% of their body weight at 16:00 per day. Throughout the trial, the temperature was controlled in 20 ± 1°C, and dissolved oxygen concentration ranged from 8.5 to 9.5 mg/L. Moreover, concentration of total ammonia nitrogen was required to be less than 0.5 mg/L, nitrites concentration less than 0.05 mg/L. Additionally, during the 42 days culture experiment, 90% of the total volume of the water in each tank was replaced daily.

### 2.3. Sample Collection

Fish were starved for 24 h before sampling. After anesthesia in a bath (20 mg/L MS‐222) for 60 s, fish were weighed and measured to determine growth performance [37]. Blood was collected from the caudal vein and replaced at 4°C overnight. Then, serum was obtained by refrigerated centrifugation (4°C, 5 min, 2500 g) for biochemical detection. The visceral mass and liver were weighed, and intestine, liver, gill, and kidney were subsequently removed and rapidly stored at −80°C for further investigation. Besides, tissue of intestine, liver, gill, and kidney by four fish in each group were fixed in 4% paraformaldehyde for histological analysis.

### 2.4. Histological Analysis

Fixed samples were processed by Servicebio (Wu han) and treated with ethanol, xylene, and toluene for dehydration and clearing. Then they were embedded in paraffin, sectioned, and stained with hematoxylin and eosin (H&E) for microscopic examination of tissue structure. The samples were photographed under a microscope (BX51, Japan) and the length, width of the intestinal villi, and the number of goblet cells within them were analyzed using ImageJ software.

### 2.5. Gut Microbiota Analysis

For each group, four intestinal tissue samples of fish were selected for the analysis of gut microbiota analysis. DNA was purified from the intestinal contents using the E.Z.N.A. Soil DNA Kit (Omega Bio‐tek, Norcross, USA). The V3–V4 regions of the bacterial *16S rRNA* gene were PCR‐amplified with barcoded primers, and the purified amplicons were sequenced using the Illumina NextSeq 2000 platform. After quality filtering and merging by fastp and FLASH, chimeras were removed using UPARSE v7.1. OTUs were classified using the RDP classifier (v2.11) against the Silva 16S rRNA database (v138). Then, the composition of species in different groups was analyzed by Parksono Genomics Cloud (https://www.genescloud.cn), and a comparative analysis of the dominant bacterial strains was conducted.

### 2.6. Growth Performance Analysis

The indices of growth performance include survival rate (SR), weight gain (WG), specific growth rate (SGR), condition factor (CF), hepatosomatic index (HIS), and visceral index (VSI). The calculation formulas for these were as follows:

SR%=100×final fish number/initial fish number,

WG%=100×final body weight - initial body weight/initial body weight,

SGR%/day=10042×ln final weight - ln initial weight/day,

CF%=100×wet body weight, g/body length, cm3,

HIS%=100× wet liver weight/wet body weight,

VSI%=100× wet visceral weight/wet body weight.

### 2.7. Biochemical Assays

Detection adopted commercial kits from Nanjing Jiancheng Bioengineering Institute (NJJC) and Beijing Solarbio Science & Technology Limited Liability Company (abbreviate Solarbio). In specific, indices like glucose (A154‐1‐1), amylase (C016‐1‐2), HK (A077‐4‐1), PFK (A129‐1‐1), PK (A076‐1‐2), PEPCK (A131‐1‐1), LDH (A020‐2), LD (A019‐2‐1), glycogen (A043‐1‐1), and MDA (A003‐1) were determined with NJJC commercial kits; G6P (BC3325), G6PDH (BC0260), SOD (BC0170), GSH‐Px (BC1190), T‐AOC (BC1315) were tested with Solarbio commercial kits. Contents or enzymes activity of this were read at diverse absorbance in a microplate reader, respectively. According to the corresponding commercial kits, the total protein concentration (A045‐4‐2) in each tissue sample was also determined to normalized the results. Experimental procedures refer to the instructions of the commercial kits. The freezing point osmotic pressure detector (Fiske Micro‐Osmometer Model 210) was used to detect the serum osmotic pressure.

### 2.8. Gene Expression Analysis

Total mRNA from the liver was extracted by Trizol reagent (Takara, Dalian, China) and reverse transcribed into cDNA via a reverse transcription kit (RR047, Takara, Japan) for qPCR template. The sequence of primers was listed in Table 2. In addition, *rpl13a* and *b2m* were used as the standard gene and were proved to be stably expressed in various organs. The expression of related genes was calculated by the 2^−ΔΔCt^ method.

**Table 2:** Primer pair sequences of the genes used for real‐time PCR (qPCR).

Gene	Direction	Primer sequence (5’‐3’)	Size (bp)	GenBank accession number
*ghra*	Forward	CATTCCGATGTTGGTGG	17	XM_044196105.1
	Reverse	TGGTGGGTGAGTCTTTCTTA	20	—
*insra*	Forward	CCAAGGTATGTATGGGTCTG	20	XM_044221015.1
	Reverse	TGTCAGGTAGCCAGTAATCTC	21	—
*insrb*	Forward	GACCCGTCGTCATACTCC	18	XM_044200278.1
	Reverse	TTCTTGCCACCGTTCAT	17	—
*glut2*	Forward	CTGCTGGACTGCTGATG	17	XM_044167564.1
	Reverse	TCTGTAAGCCTTGGGTG	17	—
*cs*	Forward	GACCCGTCGTCATACTCC	19	XM_044212662.1
	Reverse	CGATAAGATTCGTCCGTTTT	20	—
*gys2*	Forward	TGCCGATGGACTACGAG	17	XM_044185960.1
	Reverse	TGAAGCAGCCGAAACCT	17	—
*pygl*	Forward	TGCCGATGGACTACGAG	17	XM_044165888.1
	Reverse	CATGAAGCAGCCGAAAC	17	—
*rpl13a*	Forward	ATCTCCGGCAACTTCTATCG	20	XM_044166826.1
	Reverse	GCGCTTCCTCTTGTCATAGG	20	—
*b2m*	Forward	CCCTGACATCACCATCCA	18	XM_044185888.1
	Reverse	CCCAGGCGTAGTCTTTAGT	19	—

### 2.9. Data Analysis

SPSS 25 software was used for the statistical analysis. All the data are expressed as the mean ± standard error of the mean (SEM). Normality and homoscedasticity assumptions were checked before analysis. Two‐way ANOVA was used to analyze the significance of the main influences of carbohydrate level and water environment. One‐way analysis of variance, followed by Duncan’s multiple comparison test, was subsequently used to analyze all the data. For all the data, *p*  < 0.05 indicated statistical significance.

## 3. Results

### 3.1. Growth Performance

**Figure 1:**
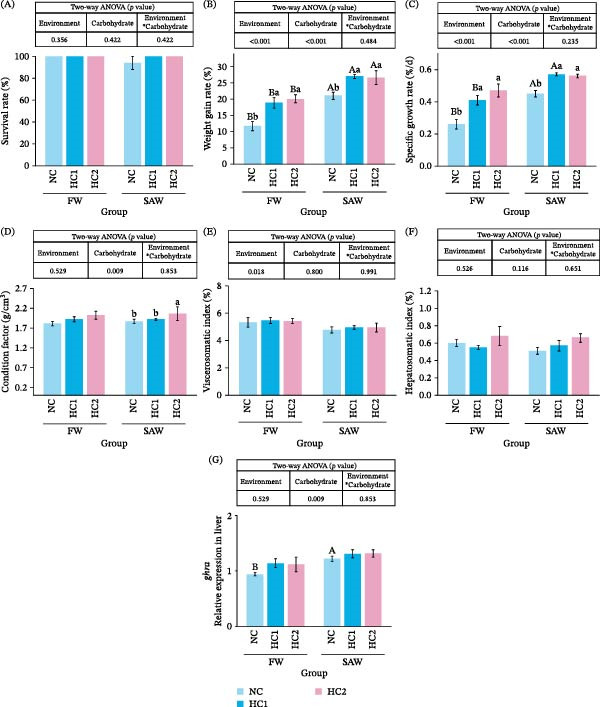
The growth parameters of mandarin fish cultured at different water environments and diverse carbohydrate levels. (A) Survival rate, (B) weight gain rate, (C) specific growth rate, (D) condition factor, (E) vicscerosomatic index, (F) hepatosomatic index, and (G) *ghra* relative expression in liver. Data were subjected to two‐way ANOVA analysis. Various capital letters on columns labeled significant difference between saline‐alkali water and fresh (*p*  < 0.05). Lowercase indicated the significant difference among diverse carbohydrate levels (*p*  < 0.05).

The death of fish only occurred in NC under saline‐alkali water, whose SR was 94.00 ± 6.00%. Nevertheless, there was no significant difference among the six groups (Figure 1A). Moreover, there was a significant increase in WG and SGR when fish were fed with higher carbohydrates in meals or bred in saline‐alkali water (Figure 1B,C). Furthermore, fish had a better CF under saline‐alkali with the highest carbohydrate fed (Figure 1D). However, VSI and HSI were not influenced by the change of environment or carbohydrate level (Figure 1E,F). In addition, the relative expression of *ghra* increased significantly in SAW + NC groups compared to FW + NC.

### 3.2. Antioxidant Status in Liver

**Figure 2:**
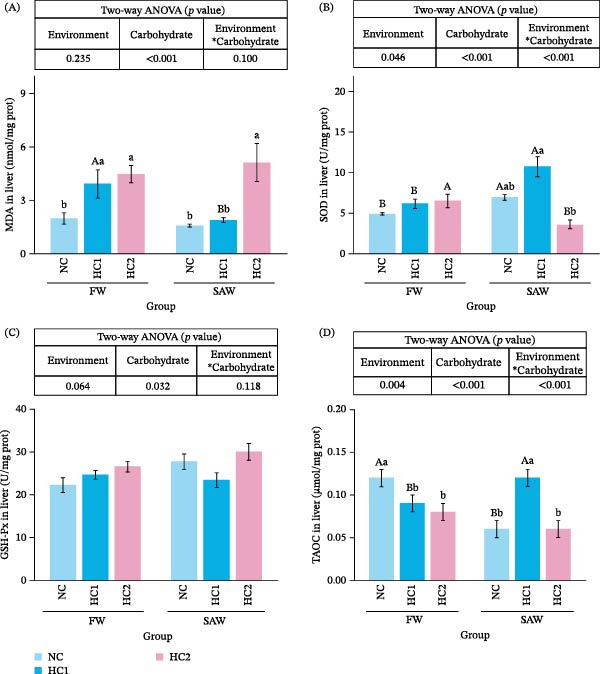
Antioxidant indices in liver among six groups. (A) MDA, (B) SOD, (C) GSH, and (D) T‐AOC in liver. Various capital letters on columns labeled significant difference between saline‐alkali water and fresh (*p*  < 0.05). Lowercase indicated the significant difference among diverse carbohydrate level (*p*  < 0.05).

Research indicated that a diet with more than 13% carbohydrate could lead to a significant accumulation of MDA in the liver from freshwater. However, in saline‐alkali water, there was no significant difference of MDA content between the SAW + HC1 group and the SAW + NC group, only a discrepancy in the SAW + HC2 group. (Figure 2A). SOD activities didn’t display significant differences in FW groups, whereas it demonstrated ascendance in the HC1 group and decline in the HC2 group from SAW groups (Figure 2B). There was not a conspicuous discrepancy of GSH activities among these groups (Figure 2C). TAOC decreased significantly with the increase of carbohydrate level; however, it soared prominently in SAW + HC1 compared to SAW + NC and SAW + HC2 (Figure 2D).

### 3.3. Tissues Morphology Analysis

To investigate the health status of tissue, histomorphology was observed, including the intestine, liver, gill, and kidney. The length and width of intestinal villi were significantly increased in high‐carbohydrate diets. Additionally, compare to FW groups, more goblet cells were observed in the SAW group (Figure 3A). Liver sections showed SAW groups performed better in tissue integrity. However, cytoplasmic vacuolation was exacerbated and intercellular space enlarged with more carbohydrate intake still (Figure 3B). Moreover, during high‐carbohydrate, gill lamellae and the external epithelial layer would become thicker and more swollen in fresh water. In contrast, except possessing more chloride cells, the morphology of gills in SAW groups seemed to be becoming better then worse with the increase of carbohydrate (Figure 3C). Observation of kidney sections demonstrated similar consequences like cytoplasmic vacuolation aggravation and hypertrophy of glomeruli performed more swollen in saline‐alkali water (Figure 3D).

Figure 3Tissue morphology observation of intestine, liver, gill, and kidney. Tissue sections were stained with H&E and observed under an optical microscope. (A) Images of intestinal sections (scale bars, 100 mm) and quantitative analysis of the length and width of the villi and the number of goblet cells in the intestine. Various capital letters on columns labeled significant difference between saline‐alkali water and fresh (*p*  < 0.05). Lowercase indicated the significant difference among diverse carbohydrate level (*p*  < 0.05). (B) Images of liver sections (scale bars, 50 mm). CV (cytoplasmic vacuolation), IS (intercellular space). (C) Images of gill sections (scale bars, 50 mm). (D) Images of kidney sections (scale bars, 50 mm). GC, goblet cell; GL, gill lamella; CC, chloride cell; EEL, external epithelial layer GR, glomerulus; CV, cytoplasmic vacuolation; GH, glomerulus hypertrophy.

**Figure:**
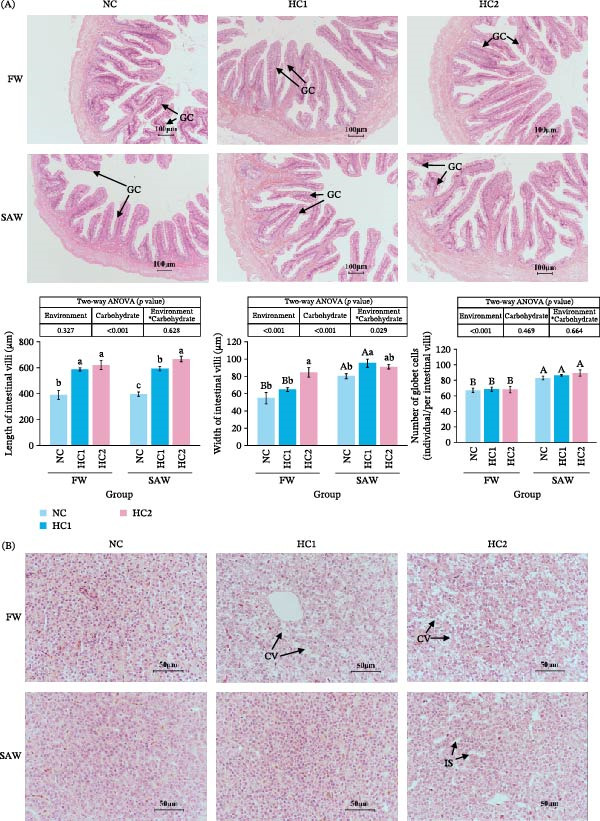

**Figure:**
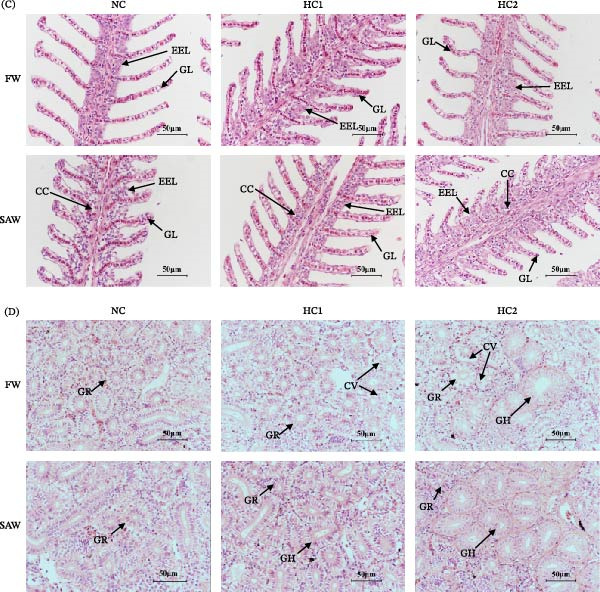

### 3.4. Process of Carbohydrate Digestion

**Figure 4:**
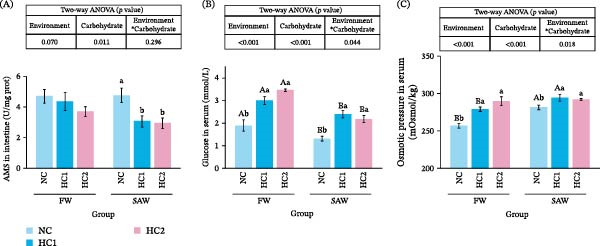
Indices of physiological phenotype under carbohydrate digestion. (A) AMS in intestine, (B) glucose in serum, and (C) osmotic pressure in the serum. Various capital letters on columns labeled significant difference between saline‐alkali water and fresh (*p*  < 0.05). Lowercase indicated the significant difference among diverse carbohydrate levels (*p*  < 0.05).

AMS in the intestine showed descendance with carbohydrate intake, particularly in SAW groups (Figure 4A). Blood glucose in the HC1 group ascended significantly compared to NC group, but had not a further rise in HC2 group (Figure 4B). Besides, fish performed higher osmotic pressure when it fed with more carbohydrate or bred in saline‐alkali water, especially in comparison between NC groups and HC1groups (Figure 4C).

### 3.5. Analysis of Changes in Gut Microbiota

**Figure 5:**
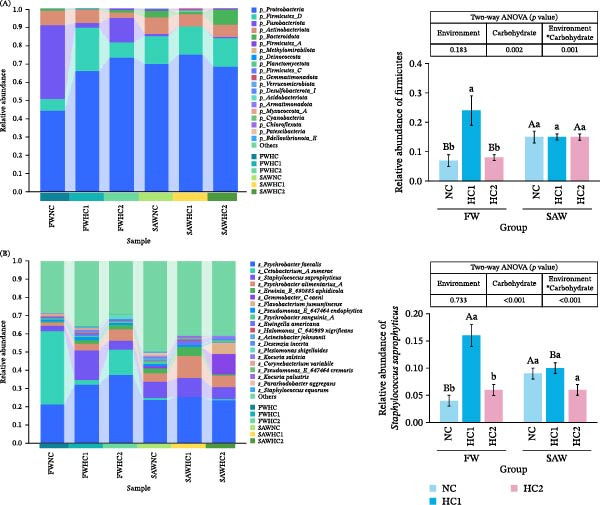
The abundance expression of gut microbiota among different groups at different classification levels. (A) Comparison of the abundance of intestinal microbiota from phylum level, especially in Firmicutes for differential analysis. (B) Comparison of the abundance of intestinal microbiota from species level, especially in *Staphylococcus saprophyticus* for differential analysis. Various capital letters on columns labeled significant difference between saline‐alkali water and fresh (*p*  < 0.05). Lowercase indicated the significant difference among diverse carbohydrate level (*p*  < 0.05).

Compared to the FW group, the abundance of the intestinal Firmicutes increased significantly in saline‐alkali water. In freshwater, FW + HC1 group showed a marked increase compared to the FW + NC and FW + HC2 (Figure 5A). Moreover, the abundance of Proteobacteria were raised in HC1 and HC2, in contrast to NC in freshwater. Meanwhile, it demonstrated higher abundance in saline‐alkali water (*p*  < 0.05). In the species level, the abundance of *Staphylococcus saprophyticus* performed rise in the HC1 group, especially from freshwater, and dropped in the HC2 group. Besides, the SAW + NC group exhibited a higher *Staphylococcus saprophyticus*’s abundance than FW + NC group (Figure 5B).

### 3.6. Relative Receptor of Glucose Metabolism in Liver

**Figure 6:**
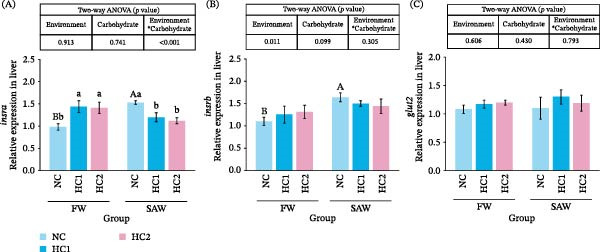
Relative expression of receptors related to glucose metabolism in liver. (A) *insra*, (B) *insrb*, and (C) *glut2* relative expression in liver. Various capital letters on columns labeled significant difference between saline‐alkali water and fresh (*p*  < 0.05). Lowercase indicated the significant difference among diverse carbohydrate levels (*p*  < 0.05).

After testing some receptors relevant to glucose regulation and transportation, we found that insulin receptor A is more sensitive than receptor B, whose relative expression is correlated with the change of carbohydrate level (Figure 6A,B). Although saline‐alkali water significantly promoted expression of *insra* and *insrb*, it demonstrated a different tendency with the increase of carbohydrate intake. During this period, the relative expression of *glut2* didn’t show significant discrepancy (Figure 6C).

### 3.7. Glycometabolism in Liver

**Figure 7:**
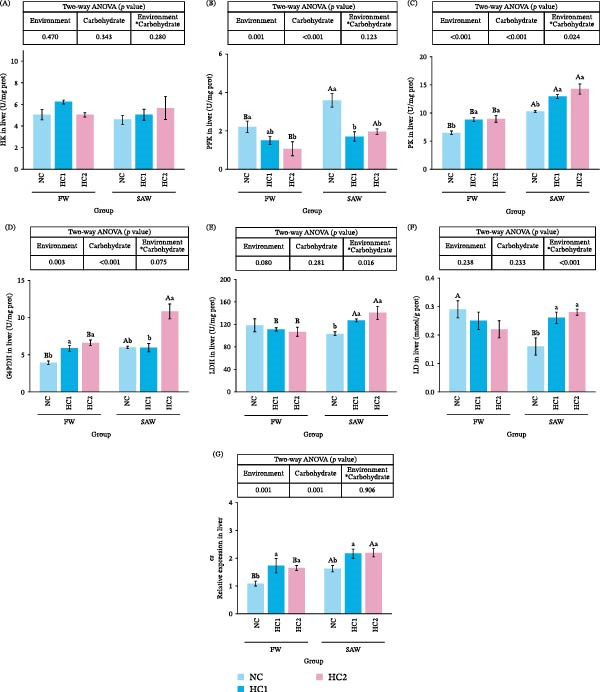
Enzymes activity and relative expression of genes in glycolysis, the pentose phosphate pathway, and the TCA cycle. (A) HK, (B) PFK, (C) PK, (D) G6PDH, (E) LDH, (F) LD, and (G) *cs* in liver. Various capital letters on columns labeled significant difference between saline‐alkali water and fresh (*p*  < 0.05). Lowercase indicated the significant difference among diverse carbohydrate level (*p*  < 0.05).

To investigate glucose metabolism in the liver, we detected enzymes and genes in glycolysis and the pentose phosphate pathway. The enzyme activity of HK didn’t show a difference among diverse groups (Figure 7A). Moreover, PFK, the crucial enzyme in glycolysis, performed a negative response with an increase of carbohydrate (Figure 7B). However, most enzymes, such as PK, G6PDH, and LDH, demonstrated positive responses of addition in carbohydrates and were conspicuously promoted by saline‐alkali water (Figure 7C–E). LD dropped significantly when fish were cultivated in saline‐alkali water with control carbohydrate. With the rise of carbohydrate levels, LD ascended dramatically in SAW groups (Figure 7E). Data on relative expression of *cs* illustrated it positively responded to the change of environment and carbohydrate (Figure 7F).

### 3.8. Glycogen Metabolism in Liver

**Figure 8:**
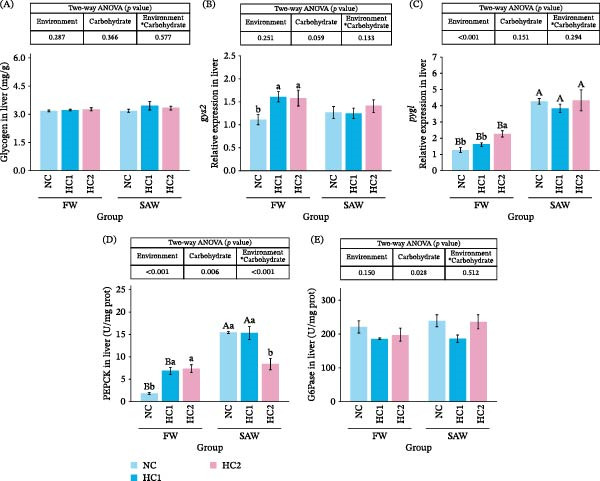
Relative expression of genes and enzymes activity and in glycogen metabolism and the gluconeogenic pathway. (A) Glycogen, (B) *gys2* relative expression, (C) *pygl* relative expression, (D) PEPCK, and (E) G6P in liver. Various capital letters on columns labeled significant difference between saline‐alkali water and fresh (*p*  < 0.05). Lowercase indicated the significant difference among diverse carbohydrate levels (*p*  < 0.05).

The results showed that there was no significant difference in glycogen content among the different groups (Figure 8A). In freshwater, the expression of the glycogen synthesis gene *gys2* increased significantly under high‐carbohydrate diet. Compared to the freshwater group, the expression of the glycogenolysis gene *pygl* in saline‐alkali water showed a significant increase (*p*  < 0.05). In freshwater, the expression of this gene was also related to the level of carbohydrate intake (Figure 8B,C). Additionally, among the two key enzymes for gluconeogenesis, PEPCK was more sensitive to changes in carbohydrate levels than G6Pase. It exhibited higher activity in saline‐alkali water (except for the SAW + HC2 group), and its expression was significantly upregulated in freshwater due to the increase in carbohydrate intake levels (Figure 8D,E).

## 4. Discussion

Previous studies indicated that saline‐alkali stress exerted negative effects on fish growth and health mostly [7–12]. In this study, fish only cultivated in the SAW + NC group appeared to death, which indicated saline‐alkali environment exerted a certainly negative impact on the growth of mandarin fish to some extent without any additional intervention. However, other scholars pointed that freshwater fish cultured in low salinity exhibited enhanced growth performance [38, 39]. From research, fish cultured in saline‐alkali water displayed better growth performance in SGR and WG, which was consistent with the latter. Among some carnivorous fish species, like Atlantic salmon and rainbow trout, environmental stresses could trigger the upregulation of the insulin‐growth hormone‐insulin‐like growth factor pathway, which promoted amino acid absorption and protein synthesis as well as assisted in environmental adaptation by facilitating osmoregulation [40, 41]. In this study, elevated insulin and growth hormone receptor (*insra* and *ghra*) expression was observed in the liver of fish exposed to saline‐alkali water. It confirmed that mandarin fish might enhance environmental adaptability and improve its growth by regulating the insulin‐related growth pathway.

As a vital substrate of nutrition, carbohydrate played a protein‐sparing role in aquatic species [22, 23, 42]. From the current study, a 13% carbohydrate diet significantly improved the growth performance of mandarin fish in both freshwater and saline‐alkali water. However, a higher intake of carbohydrates did not further promote the growth of mandarin fish, which might be ascribed to limited capacity of carbohydrate utilization as well as the growth inhibition caused by the accumulation of lipid peroxides produced in the process of glycometabolism [43–46].

MDA, as one of the final products of lipid peroxidation, was used to assess the level of lipid peroxidation and the degree of oxidative stress. During the process of glucose metabolism, the autoxidation of glucose could lead to the accumulation of MDA, thereby damaging the animal’s antioxidant system, which was manifested as the decrease in SOD and TAOC. [47, 48]. In this study, while carbohydrate supplementation could significantly promote the growth of mandarin fish, it also aggravated MDA accumulation, impaired antioxidant capacity and occurred as exacerbated cytoplasmic vacuolation, intercellular space enlargement, renal cell hypertrophy, and gill epithelial hyperplasia in tissue. However, a 13% carbohydrate diet effectively alleviated these damages under saline‐alkali water. Previous studies indicated that proper carbohydrate supplementation could promote antioxidant capacity and supply energy for osmotic adjustment under saline‐alkali stresses, thereby maintaining physiological homeostasis [49]. Results illustrated that it is more conducive to maintain oxidation balance with proper carbohydrate supplementation in saline‐alkali water for carnivorous fish, mandarin fish. However, the vigorous glycometabolism process under a high‐carbohydrate diet is also highly prone to triggering the generation and accumulation of reactive oxygen species. On the one hand, this will induce the occurrence of lipid peroxidation, leading to the accumulation of MDA [47, 48]. On the other hand, it will damage the antioxidant system and reduce the efficiency of MDA clearance [50]. From the significant accumulation of MDA and the decreased antioxidant capacity observed in the SAW + HC2 group, it could be seen that even in a saline‐alkali environment, excessive carbohydrate supplementation would also impose a metabolic burden for mandarin fish.

In regard to carbohydrate digestion and absorption, some scholars considered amylase a crucial factor which was affected by salinity and carbohydrate level [31, 51, 52]. However, others reported it was subtle and responded in carnivorous species [27, 28, 53] or even suppressed by carbohydrate addition [54, 55]. During our research, a high‐carbohydrate diet caused a descendance in amylase activities, and long‐term saline‐alkali stress exacerbated this trend, which was in accord with the results that exposure to salinity or an alkaline environment could exert a negative impact on the activity of amylase [56]. The composition and abundance of gut microbiota were believed to be closely related to the physiological functions of the host [57]. Moreover, bacteria belonging to the Firmicutes phylum were particularly adept at decomposing complex carbohydrates such as starch [58]. Furthermore, studies had shown that *Staphylococcus saprophytic*, as a member of the Firmicutes family, could play a role in reducing triglycerides and improving insulin resistance [59]. In our research, fish in saline‐alkali water exhibited higher abundance in Firmicutes and *Staphylococcus saprophytic* contrast to in freshwater. Moreover, a 13% carbohydrate diet in freshwater significantly increased the abundance of both. In combination with previous studies, it was possible that in a high‐carbohydrate diet, the decomposition and utilization of carbohydrates by mandarin fish might rely more on the role of the gut microbiota rather than its own amylase. The intestine was also involved in the digestion and absorption of nutrients as well as the osmotic regulation of fish [38]. Additionally, the length of intestinal villi was a vital index reflecting absorption capacity [60, 61]. From our observation, intestinal villi were significantly lengthened with carbohydrate addition to meet the requirement of absorption, and the number of goblet cells conspicuously surged in saline‐alkali water to protect intestinal epithelium from alkali‐induced toxicity by secreting more mucus [62]. These changes revealed the limited capacity of amylase in mandarin fish for carbohydrate digestion. However, it still met the requirements of absorption and utilization by altering intestinal structure. Additionally, there might be other reasons that truly affected the improvement of carbohydrate utilization and its metabolism in mandarin fish.

Although insulin and its receptors were crucial to glucose metabolism [29, 63], carnivorous fish possessed limited secretory capacity or receptor sensitivity [64, 65]. In the current study, the expression of two types of insulin receptors, *insra* and *insrb*, in the liver of mandarin fish was promoted by saline‐alkali stresses. Additionally, *insra* was more sensitive to the change of carbohydrate than *insrb*, which was consistent with research in zebra fish [66]. In humans, the liver could prioritize over 50% of the ingested glucose by storing in the form of glycogen, which was for future use and not affected by insulin levels [67]. In our research, the level of insulin receptors in saline‐alkali water decreased as the carbohydrate level rose, despite the fact that there was the same intense metabolic demand as in fresh water. In contrast to cultivation in fresh water, mandarin fish might be more inclined to metabolize carbohydrate through noninsulin‐dependent pathways in the liver when facing adversity. GLUT2 was an important subtype of the glucose transporter family, which played a critical role in bidirectional transportation of glucose between the liver and blood [68, 69]. In herbivorous and omnivorous fishes, like Chinese longsnout catfish, gibel carp and grass carp, the expression of *glut2* was enhanced with the growing carbohydrate intake or glucose injection [70, 71]. In contrast, there was not a significant difference in the expression of *glut2* among experimental groups in the current study, which illustrated that enhancing glucose transport might not be the primary approach for mandarin fish to promote glucose metabolism. Additionally, the glucose transport capacity which did not match the metabolic requirements of carbohydrates might also lead to the accumulation of blood glucose.

Glucose metabolism was of vital importance for the osmoregulation of fish [12, 72]. Not only can its metabolic products directly participate in regulating osmotic pressure, but the ATP produced during this process was also the main energy source for fish to cope with the hypertonic stress in saline‐alkali environments [73–75]. Additionally, structures within gill filaments also appeared to changes, such as the proliferation of chloride cells and the formation of multicellular complexes, in order to meet the requirement of osmoregulation [76–78]. In the studies, the proliferation of chlorine cells and the emergence of multicellular complexes in the SAW group indicated vigorous energy metabolism activities, which were inseparable from the regulation of osmotic pressure in saline‐alkali water. At the same time, glucose metabolism was also activated under conditions of a high carbohydrate intake or in saline‐alkali environments. However, it was restricted in high‐carbohydrate diet due to lower PFK activities. The key enzyme in the pentose phosphate pathway, G6PDH, exerted a vital impact on supplying energy in osmotic regulation and maintaining the stability of antioxidant capacity [79–82]. Additionally, it was improved by the intake of exogenous carbohydrates and more sensitive to the variation of glucose concentration [83–85]. From mandarin fish, G6PDH was stimulated under high‐carbohydrate diet and demonstrated higher activities in saline‐alkali, which satisfied the vigorous metabolic requirements under the high‐carbohydrate diets. In this condition, fructose‐6‐phosphate was metabolized through the pentose phosphate pathway to 3‐phosphoglyceric acid, which directly participates in the subsequent glycolysis process without relying on PFK to produce the precursor of 3‐phosphoglyceric acid [83, 84]. Moreover, results from *cs* and LD indicated that the product of glycolysis flowed into the TCA cycle for energy supply more instead of being stored as lactic acid.

Osmoregulation was considered a process of high energy consumption [86–88]. There were two major ways of energy supply for it, glycolysis and glycogenolysis [89, 90], in particular, glycogenolysis as prime [91–93]. A dynamic balance between glycogen synthesis and decomposition was found in our research, in which glycogen synthesized for blood glucose stability and disintegrated for energy supply. Interestingly, the expression of *pygl*, a gene related to glycogenolysis, boosted greatly in a saline‐alkali environment, which indicated a mass of energy requirement. A mechanism, called the gluconeogenesis pathway, responded to this demand [19, 94]. Research indicated that PEPCK, as a critical enzyme in the gluconeogenesis pathway, responded positively to the energy requirement, which provided additional substrates, such as glycogen, for the TCA cycle [94]. The changes among genes and enzymes revealed that the important role of the gluconeogenesis pathway during the process of adaption under a saline‐alkali environment in mandarin fish.

## 5. Conclusion

In summary, we found that appropriate saline‐alkali environment was more conducive to the growth of mandarin fish. Moreover, promoting the carbohydrate level moderately in feed could enhance the stress resistance of mandarin fish. Furthermore, in carnivorous fish, insulin regulation might not be the major strategy for glycometabolism under saline‐alkali stress, and their regulation strategies vary from fish in fresh water. Additionally, the pentose phosphate pathway might be the crucial regulatory pathway responding to glycometabolism under adverse conditions from mandarin fish.

## Funding

This study was supported by the National Key Research and Development Program of China (Grant 2023YFD2401001).

## Conflicts of Interest

The authors declare no conflicts of interest.

## Data Availability

The data that support the findings of this study are available upon request from the corresponding author. The data are not publicly available due to privacy or ethical restrictions.
